# Flavor perception and the risk of malnutrition in patients with Parkinson’s disease

**DOI:** 10.1007/s00702-018-1862-8

**Published:** 2018-02-22

**Authors:** Dareia S. Roos, Oscar J. M. Oranje, Anneleen F. D. Freriksen, Henk W. Berendse, Sanne Boesveldt

**Affiliations:** 1 Department of Neurology, VU University Medical Center, P.O. Box 7057, 1007 MB Amsterdam, The Netherlands; 2 Division of Human Nutrition, Wageningen University, P.O. Box 17, 6700 AA Wageningen, The Netherlands

**Keywords:** Parkinson’s disease, Flavor perception, Olfaction, Malnutrition, BMI

## Abstract

Flavor perception involves both olfactory and gustatory function. In patients with Parkinson’s disease (PD), hyposmia is a frequent finding, as well as an increased risk of malnutrition. We performed a pilot study to investigate the relationship between flavor perception and risk of malnutrition in PD patients. 63 PD patients participated to perform an olfactory (Sniffin’ Sticks) and gustatory (Taste Strips) task, and a questionnaire to establish nutritional risk (MUST), which includes BMI measurements. The relationship between olfactory and gustatory function and BMI was analyzed using partial correlations, corrected for disease duration, and regression analysis. Patients displayed a high prevalence of hyposmia (68.3%), and a low prevalence (6.3%) of hypogeusia. A small, but significant correlation was found between olfactory function and BMI (*r* = 0.261, *p* = 0.038), and not for gustatory function and BMI (*r* = 0.137, *p* = 0.284). Hyposmia, and not hypogeusia, may contribute to weight loss in Parkinson’s disease, and hence increase the risk of malnutrition.

## Introduction

Parkinson’s disease (PD) is associated with an increased risk of malnutrition and a lower body mass index (BMI) relative to healthy controls (Sheard et al. 2011; Chen et al. 2003; van der Marck et al. 2012; Mun et al. 2016; Uc et al. 2006; Beyer et al. 1995; Barichella et al. 2008). However, the cause of unintended weight loss in PD patients is still unclear. Potential contributing factors include disease severity and duration, higher energy expenditure caused by tremor, dyskinesia or rigidity, and differences in food intake, which may be related to medication side effects (Barichella et al. 2009; Kashihara 2006; Pfeiffer 2003; Bachmann and Trenkwalder 2006).

As sensory perception is an important determinant of food choice and intake, changes in olfactory and/or gustatory function might also contribute to unintended weight loss in PD (Spence 2015; Imoscopi et al. 2012; Gopinath et al. 2012). Hyposmia was first reported as a symptom of PD in 1975 (Ansari and Johnson 1975) and is now known to be one of the most frequently occurring non-motor features of PD that may even worsen with disease progression (Doty 2012; Berendse et al. 2011). Gustatory function can also be affected in PD patients, although the available data are less consistent (Cecchini et al. 2015).

Considering the high prevalence of hyposmia (up to 90%; Doty 2012) and the potential contribution of hypogeusia we hypothesized that a reduction in the sense of smell and/or taste would be associated with a lower BMI, and hence increase the risk of malnutrition in PD. In this brief report, we present the results of a pilot study on the relationship between the olfactory and gustatory components of flavor perception and (risk of) malnutrition in PD.

## Methods

### Participants

A total of 63 Parkinson’s patients (42 males, 21 females) participated in our study. All patients were recruited from the outpatient clinic for movement disorders at the Department of Neurology of the VU University medical center (VUmc). Each patient fulfilled the UK Parkinson’s Disease Brain Bank criteria for the clinical diagnosis of Parkinson’s disease (Hughes et al. 1992). Patients were excluded if they suffered from any other disorder known to cause loss of gustatory or olfactory function, used medication known to influence taste or smell, had a score on the Mini-Mental State Examination (MMSE) below 25 suggesting dementia, or if they were regular tobacco users. To avoid an influence on gustatory or olfactory function patients were asked not to eat or drink anything besides water for 1 h before testing and not to wear perfume.

All patients gave written informed consent. The study was approved by the Medical Ethical Committee of the VUmc.

### Gustatory function

Gustatory function was measured using the “Taste Strips” (Burghart, Germany), which consists of strips made out of filter paper, impregnated with four different tastants (each with a different quality: sweet, sour, bitter, salt) in four different concentrations (Mueller et al. 2003; Landis et al. 2009; Manzi and Hummel 2014). The strips were presented in four series of four, each series containing one of every taste quality, in ascending concentrations. The order in which the tastants were presented was randomized. The patients were asked what they tasted in a five-way forced choice (‘no taste’ was the fifth alternative), and received one point for each correct answer (0–16 for total test). After every taste strip they were asked to rinse their mouth with water.

### Olfactory function

The “Sniffin’ Sticks” test battery (Burghart, Germany) was used to measure olfactory function (Hummel et al. 1997; Kobal et al. 2000; Hummel et al. 2007). Odor identification, discrimination and detection threshold were tested separately and a total composite score (TDI; 1–48 points) was then calculated. The odorants were presented by removing the cap of the felt tip pen and then holding it under the nostrils for about 2 s at a distance of 2 cm. The exact details for testing odor identification, discrimination and detection thresholds have been reported elsewhere (Hummel et al. 2007).

### Nutritional status

Nutritional status was assessed by means of the body mass index (BMI) and the Malnutrition Universal Screening Tool (MUST) (BAPEN, Great-Britain) (Stratton et al. 2006). The MUST is a screening test for adults to determine whether they are malnourished or at risk of malnutrition. It consists of three elements: BMI, the percentage of unplanned weight loss, and presence of a current acute illness. A maximum score of two points per item can be achieved (6 in total). A score of 1 point is indicative of a medium risk, and 2 or more points signifies a high risk of malnutrition.

### Statistical analysis

The data were analyzed using SPSS 22.0 (SPSS Inc., Chicago, IL, USA). The alpha level was set at 0.05.

To compare gustatory function of our PD patients with test scores of subjects from a healthy control population, we used previously reported data, and calculated a weighted average (Landis et al. 2009). Then we tested with a one-sample *t* test for differences between the mean scores of the two groups. In a similar way, we also performed a one-sample *t* test to compare the olfactory test results from our study population with the scores of a healthy control population (Hummel et al. 2007).

The prevalence of hypogeusia was determined by the number of patients with a score below the tenth percentile on the test. For olfactory function we calculated the frequencies of hyposmia and anosmia. Hyposmia was defined as a score below the tenth percentile in comparison with values of healthy age- and gender-matched controls. Anosmia was defined as an absolute score below 16 points (Hummel et al. 2007).

We analyzed the relationship between olfactory function, gustatory function and BMI using correlations and partial correlations, with a correction for disease duration. Lastly, we used a regression analysis in which BMI was used as the dependent variable, to analyze the causal relationship between olfactory function and BMI.

## Results

The mean age of our patients was 65.9 (SD 8.5) years with a mean disease duration of 10.6 (SD 6.5) years, and a mean Hoehn and Yahr stage of 2.3 (SD 0.4; range 1–3). All of them, except one patient used dopaminergic replacement therapy (levodopa, dopamine agonist or MAO-B inhibitor).

Gustatory test scores of our PD patients did not differ significantly from the scores of the healthy population. Only four patients (6.3%; all female) suffered from hypogeusia (Table 1).

**Table 1:** One sample *t* test for gustatory and olfactory function in PD patients compared to healthy controls

	PD patients Mean (SD)	Healthy controls [13, 17] Mean	One sample *t* test (significance)
Gustatory function	9.86 (2.90)	9.67	*t* = 0.51 (*p* = 0.610)
Olfactory function: TDI	17.57 (6.45)	30.31	*t* = − 15.69 (*p* = 2.9206E−23)*
Odor detection threshold	3.04 (2.86)	7.42	*t* = − 12.17 (*p* = 4.4471E−18)*
Odor discrimination	7.57 (2.84)	10.95	*t* = − 9.45 (*p* = 1.2746E−13)*
Odor identification	6.95 (2.79)	12.28	*t* = − 15.15 (*p* = 1.6204E−22)*

*Statistical significant result

Olfactory function of the PD patients was significantly impaired compared to data from healthy controls (Table 1). Also for odor detection threshold, discrimination and identification separately the scores were significantly lower, compared to a healthy control population. 68.3% of our PD patients had an impaired sense of smell, of which 47.6% were anosmic. Odor identification was the most frequently impaired modality (85.7%), followed by discrimination (55.6%) and detection threshold (50.8%).

The mean BMI in our population was 25.04 kg/m^2^ (SD 2.95; range 20.4–31.9 kg/m^2^). Four patients (6.3%; all male) in our study population scored 1 point on the MUST (medium risk), all for unwanted weight loss between 5 and 10%. Only one of these patients was using a dopamine agonist, whereas the other three were on levodopa monotherapy. There were no PD patients with a high risk of malnutrition.

**Fig. 1:**
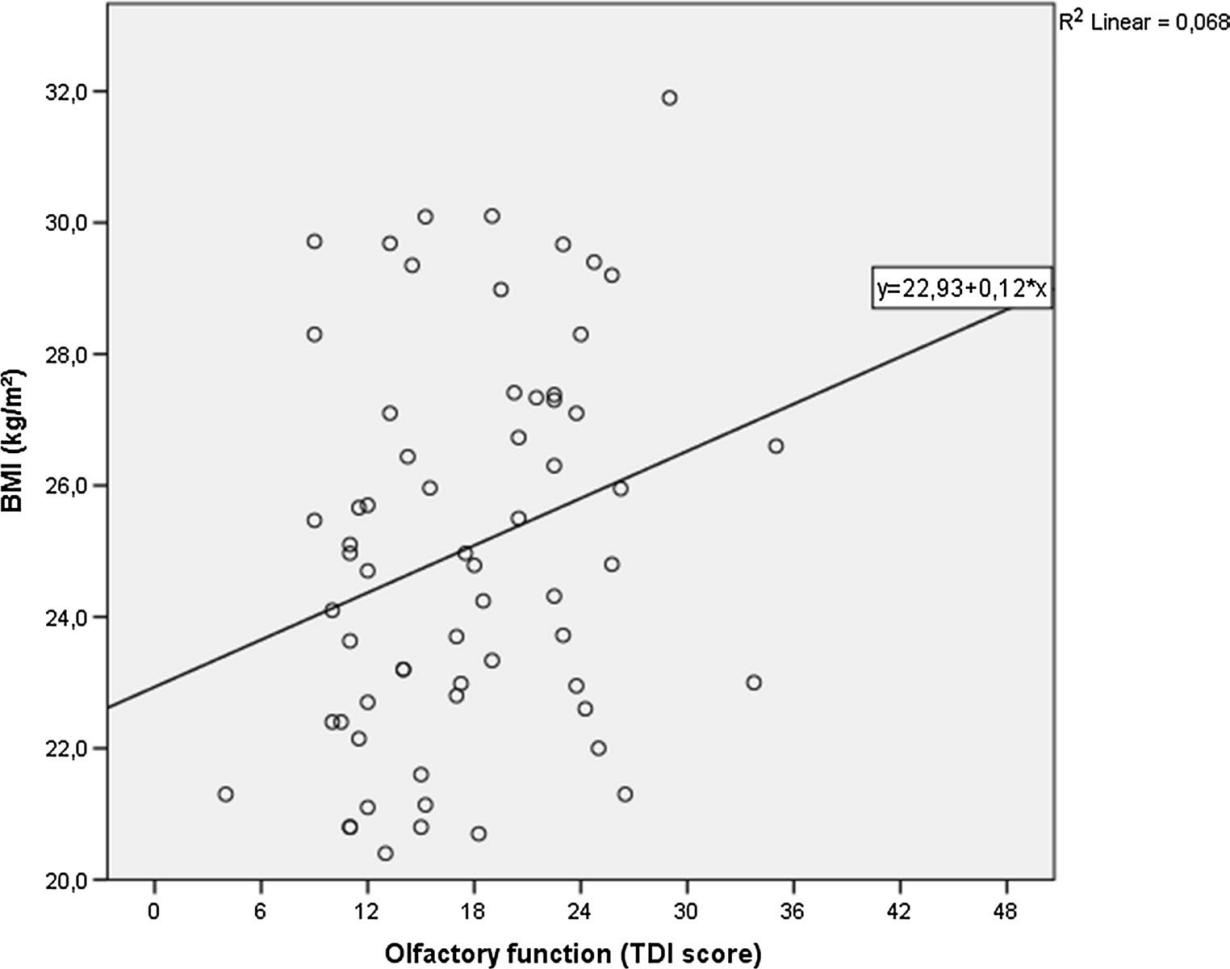
Scatterplot illustrating the correlation between olfactory function (TDI score) and BMI (kg/m^2^) in patients with Parkinson’s disease

A positive linear correlation was found between olfactory function and BMI: patients with a lower composite olfactory function (TDI) score had a lower BMI (Fig. 1). We found a significant correlation between BMI and TDI (*r* = 0.261, *p* = 0.038), even after correction for disease duration (*r* = 0.256, *p* = 0.045). Odor discrimination also had a significant correlation with BMI (*r* = 0.296, *p* = 0.019), also after correction for disease duration (*r* = 0.289, *p* = 0.023). The score on the Taste Strips was not significantly correlated to BMI (*r* = 0.137, *p* = 0.284).

There was no correlation between BMI and disease duration (*r* = − 0.135, *p* = 0.292) or age (*r* = 0.041, *p* = 0.752).

Regression analysis for olfactory function (TDI score) resulted in a *R* square of 0.07, which explained 5.3% (adjusted *R* square) of the variance of BMI (*p* = 0.04).

## Discussion

In the present pilot study we found a small, but statistically significant influence of olfactory, but not gustatory function, on BMI in non-demented PD patients: those with more severe hyposmia have a lower BMI.

In our study population, consisting of mild to moderately advanced PD patients, prevalence of hyposmia was high, confirming the results of previous studies (Doty 2012; Haehner et al. 2009). By contrast, hypogeusia was rare, suggesting that any influence of altered or diminished flavor perception on the risk of malnutrition must come from an impairment of the sense of smell, which is consistent with our results that gustatory function was not related to BMI. In some previous studies an impairment of taste in PD patients has been found; however, with a highly variable prevalence up to 20–30%, which might be due to variability in the method used for testing gustatory function (Cecchini et al. 2015). Subjective taste loss assessed by a questionnaire showed a prevalence around 13–22%, but one may question whether it is possible to distinguish between taste and smell perceptually, as both are involved in flavor perception (Pont-Sunyer et al. 2015; Deeb et al. 2010; Sienkiewicz-Jarosz et al. 2005).

The risk of malnutrition was not very high in our population: a medium risk of 6.3%. Previous studies report a prevalence of malnutrition varying between 0 and 24% in PD patients; however, using various methods to screen for malnutrition (Sheard et al. 2011). In two previous studies using the MUST in PD patients, the prevalence of a high risk of malnutrition was 5–8%, and of a medium risk 15–17%, which also relied mostly on unplanned weight loss (Jaafar et al. 2010; Barichella et al. 2013).

In spite of the fact that the mean BMI in our PD patients was within the range of the general Dutch population (NCDRF Collaboration 2016), our observation that hyposmia was associated with lower BMI values suggests that hyposmia may contribute to unintended weight loss in PD. This is in line with the results of a previous study, reporting worse olfactory function in a group of weight-losing PD patients, compared to a group of patients with a stable weight (Sharma and Turton 2012). In this study, both groups also had BMI values in the normal range. Furthermore, an association between olfactory dysfunction and malnutrition has been demonstrated in non-PD patients (Toussaint et al. 2015).

BMI is not the only factor determining malnutrition in PD. A higher risk of malnutrition was reported in 23% of a group of 61 PD patients, none of whom had a BMI < 19 (Barichella et al. 2008). Other factors associated with risk of malnutrition in PD include rigidity, dyskinesia, mood disturbances, and use of dopamine agonists (Barichella et al. 2009, 2013). These factors were not analyzed in this small-sized pilot study. However, the fact that only one out of the four patients with a higher risk of malnutrition used a dopamine agonist would seem to argue against an important influence of dopamine agonists. Furthermore, when correcting for disease duration the correlation between TDI score and BMI remained significant, which suggests it is unlikely that increasing disease severity confounded our data. Moreover, a recent meta-analysis failed to find a correlation between BMI and disease duration (van der Marck et al. 2012; Ahlskog and Muenter 2001).

Future longitudinal studies in PD, also including more advanced cases, are necessary to clarify whether the influence of olfactory function on weight is stable or progressive, and hence causes an increasing risk of malnutrition with disease progression. In such studies, the influence of other factors that may contribute to a lower BMI and malnutrition should be taken into account. These include levodopa-induced dyskinesias, cognitive decline, mood disturbances and dysphagia.

To conclude, the results of this pilot study suggest that a reduced sense of smell, but not hypogeusia, may contribute to weight loss in PD and ultimately to (risk of) malnutrition.
